# Cost–Utility Analysis of Radiation Treatment Modalities for Intermediate-Risk Prostate Cancer

**DOI:** 10.3390/curroncol28040219

**Published:** 2021-06-25

**Authors:** Najlaa Alyamani, Jiheon Song, Sasha van Katwyk, Kednapa Thavorn, Julie Renaud, Alain Haddad, Miller MacPherson, Marc Gaudet

**Affiliations:** 1 Division of Radiation Oncology, The Ottawa Hospital, University of Ottawa, Ottawa, ON K1H 8L6, Canada; jisong@toh.ca (J.S.); jurenaud@toh.ca (J.R.); ahaddad@toh.ca (A.H.); mmacpherson@toh.ca (M.M.); mgaudet@toh.ca (M.G.); 2 Ottawa Hospital Research Institute, Ottawa, ON K1Y 4E9, Canada; svank074@uottawa.ca (S.v.K.); kthavorn@ohri.ca (K.T.); 3 School of Epidemiology and Public Health, University of Ottawa, Ottawa, ON K1G 5Z3, Canada; 4 Institute for Clinical and Evaluative Sciences, Ottawa, ON K1Y 4E9, Canada

**Keywords:** cost–utility analysis, intermediate-risk prostate cancer, radiation therapy

## Abstract

Introduction: Variable costs of different radiation treatment modalities have played an important factor in selecting the most appropriate treatment for patients with intermediate-risk prostate cancer. Methods: Analysis using a Markov model was conducted to simulate 20-year disease trajectory, quality-adjusted life years (QALYs) and health system costs of a cohort of intermediate-risk prostate cancer patients with mean age of 60 years. Clinical outcomes on toxicity and disease recurrence were measured and a probabilistic sensitivity analysis was performed, varying input parameters simultaneously according to their distributions. Results: Among the six radiation treatment modalities, including conventionally fractionated intensity-modulated radiation therapy (IMRT), hypofractionated IMRT, IMRT combined with high-dose-rate (HDR) brachytherapy, HDR brachytherapy monotherapy, low-dose-rate brachytherapy monotherapy, and stereotactic body radiotherapy (SBRT), SBRT was found to be more cost-effective when compared with LDR-b and other treatment modalities, resulting in an incremental cost–utility ratio of $2985 per QALY. Conclusions: Stereotactic body radiotherapy is the most cost-effective radiation treatment modality in treatment of intermediate-risk prostate cancer, while treatment toxicity and cost data are the key drivers of the cost–utility. Further work is required with long-term follow-up for SBRT.

## 1. Introduction

Prostate cancer continues to be the most prevalent non-cutaneous cancer in males, and the third most common cause of cancer deaths in Canada [1]. In intermediate-risk prostate cancer, which accounts for approximately one-third of all prostate cancer, treatment options have evolved significantly, and different radiation therapies are available to patients. Currently, there is a high level of evidence on the effectiveness of radiation therapy in improving local control, biochemical control and overall survival when used in this group of population [2]. However, several new treatment modalities have emerged within the radiation treatment options in the last decades, but all have relatively equivalent survival outcomes [2,3,4,5]. Therefore, treatment choice is often driven by differences in patient and provider preferences that are largely part based on treatment time, cost and toxicity profile.

The recent decrease in the mortality rate of prostate cancer, in addition to this evolution in radiotherapy techniques, has added an economic burden to the Canadian health care system [6]. In 2012 alone, cancer care cost has risen to $7.5 billion compared to $2.9 billion in 2005 [7]. The 5-year mean cost per prostate cancer patient was estimated to be $30,322 ($30,308–30,336) while the mean lifetime (25 years) net cost was $79,147 ($79,110–79,181) in 1997–2007 [8]. However, these data only consider direct costs and do not account for the overall effect on the quality of life associated with concerning the cost.

A careful assessment of the economic burden, in which variable costs exist with different radiation modalities in treating prostate cancer, is thus an important step for policymakers in their decision-making, where the treatment modality associated with the most cost-effectiveness is evaluated and selected. Most data currently available on the cost analyses of localized prostate cancer treatment are based on the American health care system [9,10,11]. However, the American system bears little resemblance to the Canadian or European models. To our knowledge, a study conducted by Helou et al. is the only study available to date that has evaluated such matter from the Canadian perspective [12]. The authors conducted a cost–utility analysis comparing two radiation techniques, stereotactic body radiotherapy (SBRT) versus low-dose-rate (LDR) brachytherapy monotherapy, in low-risk prostate cancer. The study determined that at the willingness-to-pay threshold of $50,000 Canadian dollars (CAD), SBRT was found to be associated with a higher level of both cost-effectiveness and lower lifetime costs compared to that of LDR monotherapy. However, the study population was restricted to patients with low-risk prostate cancer, where only two radiotherapy techniques were compared. A more comprehensive cost-effectiveness study comparing multiple radiation techniques and schedules on different prostate cancer patient groups is therefore necessary.

In our study, we aimed to perform a cost–utility analysis of radiation modalities currently used in the treatment of an intermediate-risk prostate cancer from Ontario’s provincial health care system perspective and provide a more comprehensive overview for this group of patients.

## 2. Materials and Methods

The model simulated a cohort of male patients with a mean age of 60 years who have been diagnosed with intermediate-risk prostate cancer [13]. The following treatment modalities are in use at Canadian centres either routinely or under clinical trials: conventionally fractionated intensity-modulated radiotherapy (cfIMRT) to 78 Gy in 39 fractions [14,15], hypofractionated IMRT (hfIMRT) to 60 Gy in 20 fractions [15], HDR brachytherapy combined with IMRT (HDR-IMRT) [16,17], HDR brachytherapy monotherapy in two fractions in one implant (HDR-b) [18,19], LDR brachytherapy monotherapy (LDR-b) [20,21], as well as SBRT in five fractions [22,23].

Evidence suggests that the long-term outcomes, including overall survival, long-term management and biochemical recurrence were non-differential across treatment modalities [2,5,24]. Therefore, the model was limited to costs and health utility outcomes from which we expect differences across the modalities would exist. Outcomes within 20 years following the index treatment were evaluated as follows: 1. Adverse events or toxicities defined as either acute if they occurred within six months of the completion of radiotherapy, or chronic if beyond six months; 2. Recurrences categorized into biochemical recurrence as per Phoenix definition [25] or clinical recurrences at local, regional or distant sites; and 3. Survival outcomes determined in biochemical disease-free survival and overall survival.

**Figure 1:**
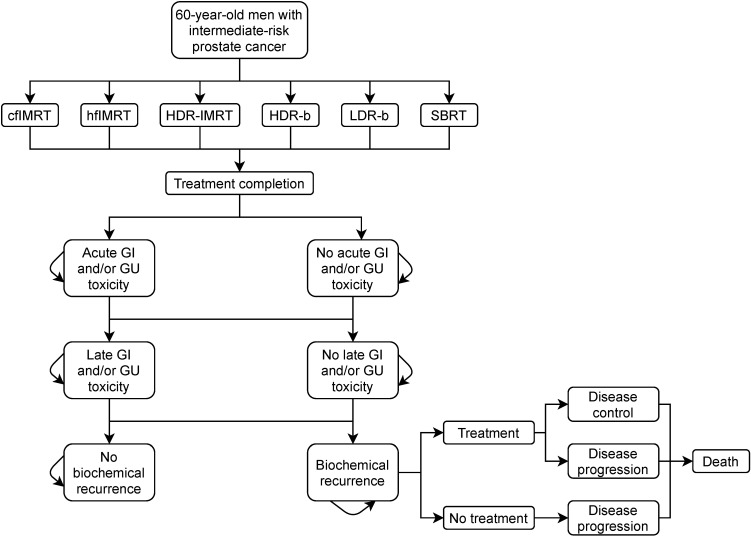
Model state transition diagram. cfIMRT = conventionally fractionated intensity-modulated radiotherapy; hfIMRT = hypofractionated intensity-modulated radiotherapy; HDR-IMRT = high-dose-rate brachytherapy combined with intensity-modulated radiotherapy; HDR-b = high-dose-rate brachytherapy monotherapy; LDR-b = low-dose-rate brachytherapy monotherapy; SBRT = stereotactic body radiotherapy; GI = gastrointestinal; GU = genitourinary.

A cohort-based Markov model was built to simulate patient distribution according to discrete health states immediately following cancer treatment until death (Figure 1). A cycle length of six-month was applied based on expert opinion that treatment strategy and general care would not significantly change in ways our model would need to differentiate in shorter time intervals. The chosen time horizon was 20 years following the index treatment. The baseline results were reported as the mean results (with 95% uncertainty variance from the mean) from the probabilistic runs. The model was run independently for each treatment modality and the associated input parameters from the published literature. Immediately following treatment, patients could suffer an acute genitourinary (GU) or gastrointestinal (GI) toxicity, remain ‘healthy’ from adverse events, or die. Following the first 6-month cycle, patients could have a recurrence event subsequently followed by a salvage treatment (cost was identical across index treatment modalities), suffer late toxicity events, remain in healthy long-term management or die. Mortality, both cancer-specific and all-cause, was based on age-adjusted general male population mortality rates across Canada, with a time-dependent relative risk factor of mortality based on published survival curves for each treatment modality. Mortality rates were assumed to not be significantly different across the modalities.

A targeted literature search was performed using PubMed free text “localized prostate cancer radiotherapy,” which yielded 9201 articles. This was narrowed down to 765 articles when limiting to prospective studies including randomized controlled trials, systematic review and meta-analysis, then 90 articles when limiting to studies that provided data on acute and late toxicities and survival outcomes. This was followed by selecting 1 to 2 most relevant trials or studies for each treatment modality, using similar or close to equivalent doses and fractionation, using similar toxicity scales as possible. Attempts were made to include Canadian studies, if available. A summary of resulted studies is available in Appendix A.

The costs of treatment modalities were based on the case-costing exercise conducted at an academic health sciences centre located in Toronto, ON, Canada. In brief, the cost of radiation treatment of prostate cancer is classified into the following two primary resources input [26]:

1. Process cost, which comprises of
  - The micro-costing supply per patient per technique used. This was calculated as per The Ottawa Cancer Centre costing of the 2018–2019 fiscal year (Appendix B)
  - The operating cost per fraction, which applies to costs borne by the hospital per fraction, and can be estimated as per Atun et al. [27]:
    - Operating cost per fraction = (“oper” + “amort” + “maint”) × 1.2 per number of fractions;
    - Where “oper” is the annual operating cost, “amort” is the amortization of the capital cost and “maint” is maintenance costs;
    - The 1.2 factor accounts for overhead;
    - A recent provincial development of the capital investment strategy was adapted for the operating cost assumptions [28];
    - The International Atomic Energy Agency (IAEA) Human Health Reports No. 13 was used to estimate the cost of the duration of a procedure such as brachytherapy [29].
2. Human resources requirement, which is comprised of
  - Physics and planning staffing levels that are estimated using Battista et al. [30], while the therapy staffing is based on Smoke et al. [31];
  - Physician remuneration which is processed primarily through the Ministry of Health schedule of benefit [32]:
    - Three remuneration models exist, among which the fee-for-service salary is the most commonly used [33].

Non-index treatment costs, including toxicity events, healthy management, treatment recurrence and its management were based on the published literature. Costs associated with management of cancer recurrence and metastasis were obtained from a Canadian cost study by Krahn et al. [34]. As there was no good data source for toxicity costs, we derived these costs from a previous costing exercise by Cooperberg et al. [9]. The setting and perspective of this publication were not perfectly matched to a Canadian health system setting and therefore beyond adjusting for inflation, a general 6-month cost estimate was applied for each parameter with an extensive variation estimate to reflect local differences in costs and friction costs associated with lower availability across different settings. This wide variance application for highly uncertain cost parameters was the best possible practice according to the Canadian guidelines [35]. All cost data were presented in 2018 Canadian dollars (CAD).

There was insufficient information on patients’ quality of life using a validated health utility score, wherein different patient outcomes, including toxicity and recurrence, are valued. Therefore, we used a more straightforward relative utility analysis that matched previous cost–utility studies on this topic [9]. Health utility values following index treatment were based on a relative dis-utility scale where post-treatment healthy status represents the best possible patient outcome and is valued at a utility score of 1. Therefore, adverse events, recurrence and subsequent treatments lead to decrement to a patient’s quality of life at a magnitude reflecting the severity of the outcome. Patient death is applied with a utility score of 0. Relative dis-utility for each patient outcome was derived from the published cost–utility studies that reported prostate cancer outcomes [9]. Our model assumes that dis-utility from an outcome is not different across treatment modalities, since there has been no evidence indicating such might be the case. Therefore, any differences in expected patient utilities were entirely driven by an event’s risk for each treatment modality.

A probabilistic analysis was performed using a Monte Carlo approach with 20,000 iterations, where input parameters were varied according to their standard errors and distributions. The mean 95% uncertainty variance from the mean of these results were reported as the baseline results of this study, following best practice [35]. A half-cycle correction was applied. An annual discount rate of 1.5% was applied to both costs and quality-adjusted life years (QALYs) as per Canadian economic evaluation guidelines. The model was developed in Microsoft Excel with Visual Basics.

The results of the model were produced based on the mean results of the probabilistic analysis. Costs and QALYs were presented as the mean expected aggregate cost or QALYs per patient. Cost–utility is measured using the incremental cost–utility ratio (ICUR) comparing six strategies; the ICUR was calculated according to the following processes:

- Modalities were ranked in terms of costs from the smallest to the largest.
- If a modality was more expensive than others or the same price but generated fewer QALYs than the preceding one, it was deemed to be “dominated” and was excluded from further analysis.
- ICURs were then calculated for each modality compared with the next most significant QALY non-dominated option. If the ICUR for a modality was lower than that of the next most effective strategy, then it was excluded by “extended dominance.”
- ICURs were recalculated, excluding any modalities subject to dominance or extended dominance.

A modality was considered more cost-effective if it was less expensive and more effective than alternative options, or if the increased cost of a modality was deemed to be justified by its increased effectiveness.

A Consolidated Health Economic Evaluation Reporting Standards (CHEERS) checklist [36] has been followed to assure the quality of the economic analysis (Appendix C).

## 3. Results

The cost–utility results are reported in Table 1. Reported results are aggregated from the probabilistic sensitivity analysis, in accordance with economic evaluation guidelines [35]. The results are presented in sequential analysis, using the lowest cost treatment as comparator. LDR-b was found to be the least costly treatment modality for the health system ($8940) when including both initial treatment and long-term outcomes. Both HDR-b and SBRT were more costly on average ($9187 and $10,048, respectively) but were associated with improved expected patient outcomes (10.63 and 11.38 average QALYs, respectively). While slightly more costly ($1.109) on average, SBRT provided more QALY gain (0.37) compared to the LDR-b, with an ICUR of $2985 per QALY. The other three comparators, cfIMRT, hfIMRT and HDR-IMRT, were found to be dominated, meaning they were more costly and less effective than LDR-b and HDR-b.

**Figure 2:**
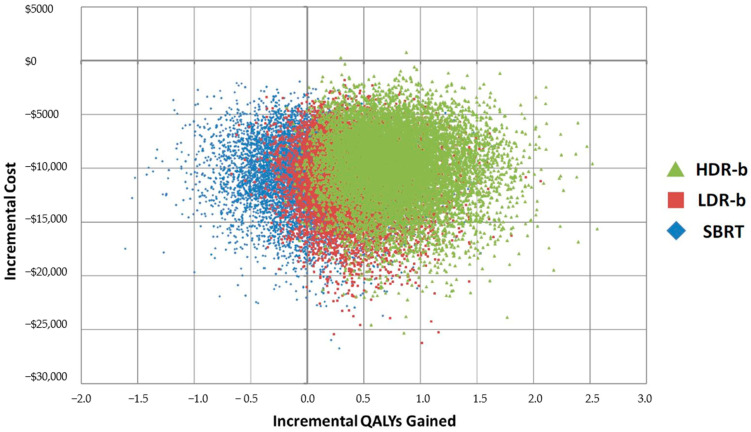
Cost-effectiveness plane, incremental results for top 3 treatment modalities. HDR-b = high-dose-rate brachytherapy monotherapy; LDR-b = low-dose-rate brachytherapy monotherapy; SBRT = stereotactic body radiotherapy; QALY = quality-adjusted life year.

**Figure 3:**
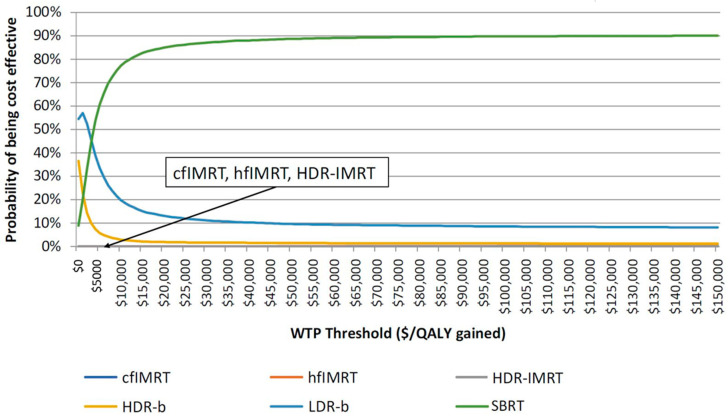
Cost-effectiveness acceptability curve of all the six radiation treatment modalities. cfIMRT = conventionally fractionated intensity-modulated radiotherapy; hfIMRT = hypofractionated intensity-modulated radiotherapy; HDR-IMRT = high-dose-rate brachytherapy combined with intensity-modulated radiotherapy; HDR-b = high-dose-rate brachytherapy monotherapy; LDR-b = low-dose-rate brachytherapy monotherapy; SBRT = stereotactic body radiotherapy; WTP = willingness-to-pay; QALY = quality-adjusted life year.

SBRT and LDR-b were found to have very close results (Figure 2), but SBRT was found to be associated with improved medium to long-term patient outcomes compared with that of LDR-b, thus allowing SBRT to be more cost-effective. Figure 3 describes the cost-effectiveness acceptability curve (CEAC) that measures the probability of each treatment to be the most cost-effective modality for a given willingness-to-pay (WTP) threshold. LDR-b was more likely to be the most cost-effective among all treatment modalities when the WTP threshold was below $5000 but was soon surpassed by SBRT as the WTP threshold increased above $5000. With the WTP threshold value of $50,000 per QALY gained, SBRT was associated with higher cost-effectiveness compared to other modalities.

## 4. Discussion

There is a lack of consensus with regard to the most appropriate radiation modality in treating intermediate-risk prostate cancer. Studies to date have demonstrated a relatively comparable rates of disease control and survival outcomes among the treatment modalities. We conducted a cost–utility analysis comparing different radiation techniques to guide our decision-making when treating this group of patients. In the absence of convincing data comparing the cost–utility studies in this area, an analysis was performed on a relative cost–utility based on the literature review of key studies on different radiation modalities.

Our study showed that SBRT is the most cost-effective compared to other techniques that were evaluated. LDR-b yielded relatively similar results to SBRT overall, likely indicating additional evidence comparing these two treatments to determine any significant differences in costs and outcomes that would change this finding. More broadly, the probabilistic analysis showed that several of these modalities were comparable, making a clear treatment decision difficult. The close overlap of results is a consequence of several shared parameter values across all modalities having a wide confidence interval associated with them, such as cost of complications and the tight marginal differences in patient outcomes across several innovative treatments, leading to very similar toxicity outcomes. More intensive surveillance of patient outcomes across each modality would improve evidence precision that may clarify treatments’ differences. Additionally, it is essential to acknowledge that some unique properties or patient requirements favor one of these treatments over SBRT under specific circumstances not captured in our model. However, it should be noted that the cost analysis of the SBRT technique used here is based on treating patients with conventional linear accelerator, which would be significantly less costly compared with other more advanced techniques, such as CyberKnife^®^ (Accuray, Sunnyvale, CA, USA). Treatment with CyberKnife^®^ in this scenario would be at least three times more costly than conventional linear accelerator-based treatments according to cost per fraction calculation at our institution.

The result of our study might not be directly comparable to the existing evidence on the cost-effectiveness of prostate cancer treatments due to differences in the study perspective and treatment modalities being considered. Cooperberg et al. [9] conducted a comprehensive lifetime cost–utility analysis of localized prostate cancer treatment, including surgery. The costs were determined from the American payer perspective, which may not be applicable to the provincial healthcare system in Ontario and most other countries. However, the results from the study showed that radiation modalities, in general, were associated with higher costs than surgical modalities. When it comes to comparing different radiation modalities, the study found that brachytherapy was the least costly among other modalities (e.g., 3D-conformal radiotherapy, IMRT alone, brachytherapy alone, IMRT combined with brachytherapy) in low- and intermediate-risk group of patients, while IMRT alone was associated with the highest cost. The result is consistent with what was found in our current analysis that both HDR-b and LDR-b were more cost-effective compared with IMRT. However, hfIMRT or SBRT techniques were not taken into account in their study. Our study findings were also in line with a recent Canadian cost study [12], which conducted a cost–utility analysis in localized prostate cancer comparing two radiation modalities (SBRT and LDR-b) in low-risk prostate cancer patients. The study concluded that SBRT is more cost-effective when compared with LDR-b. However, long-term outcomes of biochemical recurrence and survival were absent in patients treated with the SBRT case.

The major sources of uncertainty in our model were toxicity rates and the total costs associated with each treatment modality. Toxicity rates were obtained from various studies, which measured toxicity using different scales. However, attempts were made to address the concern by performing analysis based on Canadian studies that were conducted on similar patient characteristics. This is also one of the limitations of our study. The reason for high uncertainty in cost data is mostly related to two compounding factors: costs were applied according to patient outcome distribution, where uncertainty around the risk of events directly impacts the variance in the total cost estimates, and a broader variance was used to post-treatment costs themselves due to the significant variability in health systems costs across Canada following index treatment. 

We limited our study to model toxicity, recurrence in the first 20 years, and associated short-term costs and utilities based on evidence that long-term outcomes were non-differential across treatment modalities [5]. Therefore, our results should not be interpreted as such that reflect the total cost of treatment or the entirety of the quality of life of patients. Instead, our study focuses on those aspects of early-stage therapy and outcomes from which we expect differences across modalities. Such practice might be helpful in decision-making and understanding the trade-offs and relative cost–utility of one treatment over another. The utility measure reported in our study is based on a relative dis-utility score, suggesting patients who had better performance status following completion of treatment without adverse events or recurrence were scored as having ‘full’ utility (score of 1) with dis-utilities applied based on the risk of events. This suggests that the reported cost–utility measures cannot be applied to non-prostate cancer patients or compared across patients in the same manner as QALY would allow. This limitation is due to the shortage of evidence on prostate cancer patient outcomes adjusted for several factors such as patient’s age, and co-morbidity compared to non-cancer patients that estimate QALYs. Our approach, however, matches the best available previously published work on this issue [9], allowing for the best comparison and interpretation within the currently available evidence.

## 5. Conclusions

Our study is one of the first studies on comprehensive cost–utility analysis across multiple radiation treatment modalities in the Canadian context. The study demonstrated that SBRT was associated with the highest level of cost-effectiveness compared with other treatment modalities. Longer follow-up on SBRT outcomes is necessary to confirm findings of our study.

## Figures and Tables

**Table 1:** Cost-effectiveness for radiation treatment modalities.

Description	Probabilistic Averages		Incremental ^§^		Sequential Analysis
	Total Cost ($)	QALYs	Cost ($)	QALYs	ICUR ($ per QALY Gained)
LDR-b	8940	11.00	n/a	n/a	Reference
SBRT	10,048	11.38	1109	0.37	$2985 *
(Dominated treatment modalities)					
HDR-b	9187	10.63	n/a	n/a	(Dominated by LDR-b **)
hfIMRT	14,332	10.86	n/a	n/a	(Dominated by LDR-b **)
HDR-IMRT	16,939	9.95	n/a	n/a	(Dominated by HDR-b ^†^)
cfIMRT	19,903	10.59	n/a	n/a	(Dominated by HDR-b ^†^)

^§^ Any discrepancies are due to rounding. * When compared with LDR-b; ** HDR-IMRT and cfIMRT were associated with higher costs and lower effectiveness when compared with HDR-b, thus dominated by HDR-b; ^†^ HDR-b and hfIMRT were associated with higher costs and lower effectiveness when compared with LDR-b, thus dominated by LDR-b. cfIMRT = conventionally fractionated intensity-modulated radiotherapy; hfIMRT = hypofractionated intensity-modulated radiotherapy; HDR-IMRT = high-dose-rate brachytherapy combined with intensity-modulated radiotherapy; HDR-b = high-dose-rate brachytherapy monotherapy; LDR-b = low-dose-rate brachytherapy monotherapy; SBRT = stereotactic body radiotherapy; ICUR = incremental cost–utility ratio.

## Data Availability

Data is contained within the article and its supplementary material.

## Appendix A

Input parameters for the model data source. Currency in Canadian Dollar ($)

cfIMRT = conventionally fractionated intensity-modulated radiotherapy; hfIMRT = hypofractionated intensity-modulated radiotherapy; HDR-IMRT = high-dose-rate brachytherapy combined with intensity-modulated radiotherapy; HDR-b = high-dose-rate brachytherapy monotherapy; LDR-b = low-dose-rate brachytherapy monotherapy; SBRT = stereotactic body radiotherapy; GU = genitourinary; GI = gastrointestinal

**Table A1:** Transition probabilities.

	Value	Standard Error	Distribution	Source
cfIMRT				
Acute GU toxicity	0.400	0.100	β	Catton et al., 2017 [15] Peeters et al., 2006 [14]
Acute GI toxicity	0.330	0.083		
Late GU toxicity	0.080	0.010		
Late GI toxicity	0.047	0.012		
Recurrence	0.009	0.002		
hfIMRT			β	Catton et al., 2017 [15]
Acute GU toxicity	0.270	0.068		
Acute GI toxicity	0.160	0.040		
Late GU toxicity	0.022	0.006		
Late GI toxicity	0.013	0.003		
Recurrence	0.006	0.002		
HD-IMRT			β	Hoskin et al., 2012 [17] Morton et al., 2010 [16]
Acute GU toxicity	0.620	0.155		
Acute GI toxicity	0.065	0.016		
Late GU toxicity	0.272	0.068		
Late GI toxicity	0.010	0.003		
Recurrence	0.002	0.001		
HDR-b			β	Hoskin et al., 2014 [17] Morton et al., 2017 [18]
Acute GU toxicity	0.410	0.103		
Acute GI toxicity	0.010	0.003		
Late GU toxicity	0.310	0.077		
Late GI toxicity	0.030	0.007		
Recurrence	0	0		
LDR-B			β	Keyes et al., 2009 [21]
Acute GU toxicity	0.260	0.065		
Acute GI toxicity	0.110	0.028		
Late GU toxicity	0.025	0.005		
Late GI toxicity	0.009	0.002		
Recurrence	0.001	0		
SBRT			β	Katz et al., 2014 [22] Jackson et al., 2018 [23]
Acute GU toxicity	0.220	0.055		
Acute GI toxicity	0.040	0.010		
Late GU toxicity	0.084	0.021		
Late GI toxicity	0.050	0.013		
Recurrence	0	0		

**Table A2:** Costs.

	Value	Standard Error	Distribution	Source
Index treatment				
cfIMRT	$12,284	$5283	γ	Micro-costing exercise
hfIMRT	$6992	$4324		
HDR-IMRT	$7051	$8250		
HDR-b	$1642	$2151		
LDR-b	$2067	$2887		
SBRT	$3033	$2973		
Adverse events and subsequent costs				
Healthy management (annual)	$238	$368	γ	Cooperberg et al., 2013 [9] Krahn et al., 2014 [34]
GI toxicity	$300	$567		
GU toxicity	$300	$484		
Metastasis management (annual)	$17,346	$4337		
Recurrence management (annual)	$8195	$2049		

**Table A3:** Utilities.

	Value	Standard Error	Distribution	Source
Post-treatment healthy	1	n/a	β	Cooperberg et al., 2013 [9]
GI toxicity, dis-utility	−0.25	−0.06		
GU toxicity, dis-utility	−0.25	−0.06		
Recurrence with treatment, dis-utility	−0.4	0.10		
Death	0	n/a		
Discount	0.015	n/a	Normal	Cooperberg et al., 2013 [9]
Age	60	12		

## Appendix B

**Table A4:** Cost table. Currency in Canadian Dollar ($).

Item	Cost	cfIMRT	hfIMRT	HDR-IMRT	HDR-b	LDR-b	SBRT
**Machine cost**							
Planning CT scan	$451.98	$451.98	$451.98	$451.98	n/a	n/a	$451.98
Post-treatment CT scan	$451.98	n/a	n/a	n/a	n/a	$451.98	n/a
(Parameter: average fractions per patient)		(39×)	(20×)	(1×) (15×)	(2×)	(1×)	(5×)
Linear accelerator	$270.74	$10,558.95	$5414.85	$4061.14	n/a	n/a	$1353.71
CyberKnife^®^ *	$1275.45	n/a	n/a	n/a	n/a	n/a	n/a
**Personnel Fees**							
Decision to treat consult	$228.60	$228.60	$228.60	$228.60	$228.60	$228.60	$228.60
3D-treatment planning	$811.15	$811.15	$811.15	$811.15	n/a	n/a	$811.15
Interstitial source application	$223.65	n/a	n/a	$223.65	$223.65	$223.65	n/a
Transrectal ultrasound	$53.10	n/a	n/a	$53.10	$53.10	$53.10	n/a
Ultrasound tech hourly rate	$41.60	n/a	n/a	n/a	n/a	n/a	$41.60
Fiducial insertion	$85.45	n/a	n/a	n/a	n/a	n/a	$85.45
Assessment during ultrasound	$37.05	n/a	n/a	$37.05	$37.05	$37.05	n/a
(Parameter: average weekly observation visit)		(8×)	(4×)	(3×)	(n/a)	(n/a)	(2×)
Weekly observation	$37.05	$296.40	$148.20	$111.15	n/a	n/a	$74.10
(Parameter: surgical and anesthetics time)		(n/a)	(n/a)	(2.5×)	(2.5×)	(2.5×)	(n/a)
Anesthesiologist hourly rate	$90.01	n/a	n/a	$225.25	$225.25	$225.25	n/a
Anesthesia assistant hourly rate	$43.60	n/a	n/a	$108.99	$108.99	$108.99	n/a
(Parameter: OR nursing time)		(n/a)	(n/a)	(4×)	(4×)	(4×)	(n/a)
Nursing hourly rate	$39.16	n/a	n/a	$156.64	$156.64	$156.64	n/a
**Material cost**							
(Parameter: number of patients per year)		(/4846)	(/4846)	(/4846)	(/4846)	(/4846)	(/4846)
Exam room supplies	$881	$0.18	$0.18	$0.18	$0.18	$0.18	$0.18
Immobilization devices (multi use)	$2508	$0.52	$0.52	$0.52	$0.52	$0.52	$0.52
Labels	$512	$0.11	$0.11	$0.11	$0.11	$0.11	$0.11
MRI	$5647	n/a	n/a	n/a	n/a	n/a	$1.17
Nursing cart supplies	$31,927	$6.59	$6.59	$6.59	$6.59	$6.59	$6.59
Office supplies	$9375	$1.93	$1.93	$1.93	$1.93	$1.93	$1.93
Other	$9074	$1.87	$1.87	$1.87	$1.87	$1.87	$1.87
Paper	$2319	$0.48	$0.48	$0.48	$0.48	$0.48	$0.48
Patient supplies	$7596	$1.57	$1.57	$1.57	$1.57	$1.57	$1.57
Radio-opaque markers	$11,657	n/a	n/a	n/a	n/a	n/a	$48.11 ^§^
Tattoo supplies	$778	$0.16	$0.16	$0.16	n/a	n/a	$0.16
LDR brachytherapy seeds	$2573.01	n/a	n/a	n/a	n/a	$2573.01	n/a
Brachytherapy	$56,889	n/a	n/a	$156.52 *	$156.52 *	$156.52 *	n/a
Total cost		$12,360.49	$7068.19	$6638.63	$1203.05	$4228.04	$3109.28

* CyberKnife^®^ only applicable for certain institutions. cfIMRT = conventionally fractionated intensity-modulated radiotherapy; hfIMRT = hypofractionated intensity-modulated radiotherapy; HDR-IMRT = high-dose-rate brachytherapy combined with intensity-modulated radiotherapy; HDR-b = high-dose-rate brachytherapy monotherapy; LDR-b = low-dose-rate brachytherapy monotherapy; SBRT = stereotactic body radiotherapy; CT = computed tomography; MRI = magnetic resonance imaging; n/a = not applicable; OR = operating room. ^§^ Radio-opaque markers applied only to SBRT which represent 5% of all treatment modalities per year, * Brachytherapy represent 7.5% of all treatment modalities per year.

## Appendix C

**Table A5:** Consolidated Health Economic Evaluation Reporting Standards (CHEERS) checklist of the present article based on ISPOR task report [36].

Section/Item	Item	Recommendation	Reported on Page No./Line No.
Title and abstract Title	1	Identify the study as an economic evaluation or use more specific terms such as “cost-effectiveness analysis”, and describe the interventions compared.	1/2–3
Abstract	2	Provide a structured summary of objectives, perspective, setting, methods (including study design and inputs), results (including base case and uncertainty analyses), and conclusions.	1/13–27
Introduction Background and objectives	3	Provide an explicit statement of the broader context for the study. Present the study question and its relevance for health policy or practice decisions.	1/30–45 2/46–69
Methods Target population and subgroups	4	Describe characteristics of the base case population and subgroups analysed, including why they were chosen.	2/70–78
Setting and location	5	State relevant aspects of the system(s) in which the decision(s) need(s) to be made.	3/120–122
Study perspective	6	Describe the perspective of the study and relate this to the costs being evaluated.	2/66–69
Comparators	7	Describe the interventions or strategies being compared and state why they were chosen.	2/71–78
Time horizon	8	State the time horizon(s) over which costs and consequences are being evaluated and say why appropriate.	2/93
Discount rate	9	Report the choice of discount rate(s) used for costs and outcomes and say why appropriate	5/175
Choice of health outcomes	10	Describe what outcomes were used as the measure(s) of benefit in the evaluation and their relevance for the type of analysis performed.	5/178–193
Measurement of effectiveness	11a	Single study-based estimates: Describe fully the design features of the single effectiveness study and why the single study was a sufficient source of clinical effectiveness data.	NA
	11b	Synthesis-based estimates: Describe fully the methods used for identification of included studies and synthesis of clinical effectiveness data.	2–3/89–105
Measurement and valuation of preference based outcomes	12	If applicable, describe the population and methods used to elicit preferences for outcomes.	3/106
Estimating resources and costs	13a	Single study-based economic evaluation: Describe approaches used to estimate resource use associated with the alternative interventions. Describe primary or secondary research methods for valuing each resource item in terms of its unit cost. Describe any adjustments made to approximate to opportunity costs.	NA
	13b	Model-based economic evaluation: Describe approaches and data sources used to estimate resource use associated with model health states. Describe primary or secondary research methods for valuing each resource item in terms of its unit cost. Describe any adjustments made to approximate to opportunity costs.	2/89–98 3/99–105
Currency, price date, and conversion	14	Report the dates of the estimated resource quantities and unit costs. Describe methods for adjusting estimated unit costs to the year of reported costs if necessary. Describe methods for converting costs into a common currency base and the exchange rate.	4/145–156
Choice of model	15	Describe and give reasons for the specific type of decision analytical model used. Providing a figure to show model structure is strongly recommended.	2/89–98 3/99–105
Assumptions	16	Describe all structural or other assumptions underpinning the decision-analytical model.	3/120–124 4/125–144
Analytical methods	17	Describe all analytical methods supporting the evaluation. This could include methods for dealing with skewed, missing, or censored data; extrapolation methods; methods for pooling data; approaches to validate or make adjustments (such as half cycle corrections) to a model; and methods for handling population heterogeneity and uncertainty.	5/178–193
Results Study parameters	18	Report the values, ranges, references, and, if used, probability distributions for all parameters. Report reasons or sources for distributions used to represent uncertainty where appropriate. Providing a table to show the input values is strongly recommended.	Appendix A and Appendix B
Incremental costs and outcomes	19	For each intervention, report mean values for the main categories of estimated costs and outcomes of interest, as well as mean differences between the comparator groups. If applicable, report incremental cost effectiveness ratios.	5/197–208 6/209–229
Characterising uncertainty	20a	Single study-based economic evaluation: Describe the effects of sampling uncertainty for the estimated incremental cost and incremental effectiveness parameters, together with the impact of methodological assumptions (such as discount rate, study perspective)	NA
	20b	Model-based economic evaluation: Describe the effects on the results of uncertainty for all input parameters, and uncertainty related to the structure of the model and assumptions.	8/284–293
Characterising heterogeneity	21	If applicable, report differences in costs, outcomes, or cost-effectiveness that can be explained by variations between subgroups of patients with different baseline characteristics or other observed variability in effects that are not reducible by more information.	NA
Discussion Study findings, limitations, generalisability, and current knowledge	22	Summarise key study findings and describe how they support the conclusions reached. Discuss limitations and the generalisability of the findings and how the findings fit with current knowledge.	7/236–263 8/264–311
Other Source of funding	23	Describe how the study was funded and the role of the funder in the identification, design, conduct, and reporting of the analysis. Describe other non-monetary sources of support.	9/323
Conflicts of interest	24	Describe any potential for conflict of interest of study contributors in accordance with journal policy. In the absence of a journal policy, we recommend authors comply with International Committee of Medical Journal Editors recommendations.	9/324
